# Active Turnover of Heme in Hibernation Period in Mammals

**DOI:** 10.3389/fphys.2019.01586

**Published:** 2020-01-15

**Authors:** Phil Jun Lee, Namki Cho, Hee Min Yoo, Hong Pyo Kim

**Affiliations:** ^1^College of Pharmacy, Ajou University, Suwon, South Korea; ^2^Research Institute of Pharmaceutical Science and Technology, Ajou University, Suwon, South Korea; ^3^College of Pharmacy and Research Institute of Drug Development, Chonnam National University, Gwangju, South Korea; ^4^Center for Bioanalysis, Korea Research Institute of Standards and Science, Daejeon, South Korea

**Keywords:** heme biosynthesis, pre-hibernation, mammals, heme oxygenase (HO)-1, calorie restriction, sirt1

## Abstract

Heme oxygenase (HO)-1 plays an important role during hibernation by catalyzing the degradation of heme to biliverdin/bilirubin, ferrous iron, and carbon monoxide, which activates the protective mechanisms against stress. In this context, it was important to analyze the metabolic processes of heme. Nevertheless, to date, no study has approached on biosynthesis of heme. Therefore, our study aims to understand the process of heme biosynthesis, which regulates cell survival in conditions of hypothermia and calorie restriction (CR). During hibernation, the mRNA levels of enzymes responsible for *de novo* heme biosynthesis were increased in the liver tissue of a Syrian hamster model of hibernation. Moreover, heme trafficking and iron metabolism were found to be more active, as assessed based on the changes in the levels of heme transporter and ferroportin mRNA. The levels of HO-1, a powerful antioxidant, were also upregulated during hibernation. Additionally, increased levels of Sirt-1 mRNA were also observed. These enzymes are known to act as cellular metabolic sensors that activate the cytoprotective mechanisms. These results indicate that HO-1 induction, brought about by the upregulation of heme during the pre-hibernation period, may protect against external stress. Here, we describe heme catabolism during hibernation by analyzing the regulation of the key molecular players involved in heme metabolism. Therefore, this study offers a new strategy for the better regulation of intracellular heme concentrations during hypothermia and other stresses.

## Introduction

Certain mammals, including primates undergo hibernation, a state in which the animals become inactive (Dausmann et al., 2004). In small rodents, such as the Syrian golden hamster, *Mesocricetus auratus*, hibernation has two phases: torpor and arousal. During recurring torpor, animals experience low body temperature (Tb) and become inactive. During arousal, the core temperature of the animals and the metabolic rate return back to normal (normal body temperature, 37°C) (Körtner and Geiser, 2000). Food intake is stopped during hibernation, and the fat stored in white adipose tissue is used as the primary energy source. In addition to relying on fatty acid metabolism, hibernators are able to survive for 5 to 8 months by reducing their metabolic rate. The profound metabolic depression of torpor reduces energetically expensive cellular processes, such as transcription and translation, and physiological functions, such as heart rate, respiration, immune and renal function, and neural activity (Carey et al., 2003). Intriguingly, hibernators avoid cellular and tissue injury when metabolic demand is reduced.

Heme, a prosthetic group with an iron ion (ferrous) in the center of a porphyrin ring structure, is an essential co-factor for the functioning of proteins involved in basic biological processes, including respiration, signal transduction, energetic homeostasis, mRNA processing, and the control of circadian rhythms (Boon et al., 2005). For instance, heme serves as a ligand for the transcription factor Rev-erb, and plays a regulatory role by modulating the function of Rev-erb (Yin et al., 2007). Heme oxygenase (HO) catalyzes the first and rate-limiting step in heme degradation, producing carbon monoxide (CO), biliverdin, and ferrous iron (Tenhunen et al., 1968). HO exists in two isoforms, HO-1, which is inducible, and HO-2, which is constitutively expressed. The present study examines the tissue-specific responses and regulation of HO-1 in the degradation of heme in the Syrian golden hamster during induced hibernation. To date, the mechanism underlying pre-hibernation remain unclear. In this study, we investigated the enzymes triggering heme synthesis, and analyzed the expression of genes that play an important role in heme metabolism and transport, including SLC46A1, transferrin receptor (TfR), divalent metal transporter-1 (DMT1), and ferroportin.

## Materials and Methods

### Animals and Housing

All animal experiments were conducted in accordance with the Guidelines for the Care and Use of Laboratory Animals, and were approved by the Animal Ethics Review Committee of Ajou University (Permission number: 2013-0006). Syrian male hamsters (6-week-old) were purchased from animal laboratories (DBL, Yumseng, South Korea). Briefly, twelve-lined Syrian male hamsters (initial body mass 130–180 g) in each group were housed at 21°C under a 12 h light:12 h dark cycle, and given *ad libitum* access to chow until they reached a plateau weight of 220–240 g. To induce pre-hibernation, some animals were placed in a cold chamber at 4–5°C in constant darkness, which resulted in their entering torpor within several days. Animals were sampled after 5 days of continuous torpor, as indicated by a constant low core Tb of approximately 5°C (measured via a subcutaneously implanted thermal transmitter (Chayama et al., 2019). The animals were maintained under conditions of caloric restriction (∼30% lower food intake than controls) at 21°C; both experimental animals and controls were sampled on the same dates. Animals were decapitated and tissue samples were collected quickly, frozen immediately in liquid nitrogen, air-freighted to Ajou University on dry ice, and stored at –80°C until use. As reported previously, the body temperature in caloric restriction and that of its control did not change during the experiment period (Talaei et al., 2011).

### Quantitative RT-PCR

The analysis of mRNA in liver tissues was carried out by a third party (Bioneer Inc., Daejeon, South Korea) in accordance with the Minimum Information for Publication of Quantitative Real-Time PCR Experiments guidelines. The primers used have been listed below.

**Table:** 

**#**	**Gene**	**Forward sequence**	**Reverse sequence**
1	*Sirt1*	GAAAGTAAGACCAGTAGCAC	CACCTAACCTATGACACAAC

2	*TFEB*	CATTTGGTGCTAACAGCTC	TACTAAAGGCACAAAGTGG

3	*Slc46A1*	GGACCCTCTACATGAACGTG	ACCACAAAGATGGACACCAC

5	*Transferrin receptor (TfR)*	CAGTGGCTGTATTCTGCTCG	CGTAGGGTGACAGGAAGTGA

6	*DMT1*	ATCTACAAAGGGGGTGTTG	CGACCATTTTAGGTTCAGG

7	*Ferroportin*	TCGGACTGGTCTGTTCTCAG	TGGTAGTTAGGGACACTGGC

8	*HMOX2*	CATGCTTACACTCGTTACAT	GTAGAACTGCTTGAATTGCT

9	*BVR*	ACAGAAAGGGAAAGTCCTG	CCAAACTTCTCTTCTTCCAG

10	*Hsp70*	TGAGAAGTTTGTGAGTGAAG	GTCCACATAAACTTGCTTTG

11	*Hsp90*	AGGTGGTCGTATCAAACCGA	TCCAGGTGTTTCTTTGCTGC

12	*ALAS1*	AATTTACTCTGACTCTGGGA	TCAAATGCTACAATCTTGGG

13	*FECH*	GAATACTCCCAAGTGTTAGC	CTTGTTTGACTGGATGTGTG

14	*CPOX*	GAACATTTCTGTCGTTCATG	AGCTTATACCCATAGCAG

15	*UROD*	CTCGGATGAAAGCGGTGAC	CACCAAACGGGAGTGTAGTC

16	*Hemoglobin*	CACCACCAAGACCTACTTC	ATCCACACGCAGTTTGTGG

			

### Quantitation of Heme in Liver Tissue

Intracellular heme levels were measured using a heme colorimetric assay kit (BioVision, Mountain View, CA, United States). Briefly, animal tissues were lysed in lysis buffer and homogenized using sonication. Aliquots (50 μL) from each diluted sample or standard were transferred into 96-well plates prior to the addition of 50 μL of heme assay kit solution to each well. After incubation for 30 min, the absorbance was measured at 570 nm using an automated microplate luminometer (BioTek, San Diego, CA, United States).

### Western Blotting

Animal tissues were lysed with radioimmunoprecipitation assay (RIPA) buffer [1 × PBS, 1% (v/v) Non-idet P-40 (NP-40), 0.5% (w/v) sodium deoxycholate, 0.1% (w/v) sodium dodecyl sulfate (SDS), 0.1 mg/mL phenylmethylsulfonyl fluoride (PMSF), 30 μl/mL aprotinin, and 1 mM sodium orthovanadate (Na_2_VO_3_)], and homogenized by sonication (Lee et al., 2015). Total tissue lysates were centrifuged and the resulting supernatants were collected. Proteins were separated on an 8–15% SDS–polyacrylamide gel and transferred onto a polyvinylidene difluoride (PVDF) membrane. The membranes were blocked with Tris-buffered saline (TBS) containing 0.1% Tween-20 (TBST) and 5% non-fat dry milk for 1 h at room temperature, and then incubated overnight with primary antibodies prepared in TBST containing 1% non-fat dry milk at 4°C. Anti-ALAS1, anti-HMOX1, anti-Bcl2, anti-Bcl_XL_, and anti-β-actin were purchased from Santa Cruz Biotechnology (Santa Cruz, CA, United States). Anti-dinitrophenol was purchased from Abcam (Cambridge, MA, United States). Membranes were washed with TBST and incubated with goat anti-rabbit or anti-mouse horseradish peroxidase-conjugated IgG secondary antibody for 2 h. Chemiluminescence was measured using the chemiluminescence system (GE Healthcare, Piscataway, NJ, United States).

### Detection of Heme-Containing Proteins by Chemiluminescence

Heme-containing proteins were identified on PVDF membranes, as described previously, using chemiluminescence (CL), which detects the presence of heme-associated horseradish peroxidase activity (Dorward, 1993). Briefly, each protein extracted from animal tissue was loaded onto 12% gel, and then transferred to a PVDF membrane. In accordance with the manufacturer’s protocol, the membrane was exposed to a mixture of CL reagents 1 and 2 for 3 min, and developed using the CL system (GE Healthcare).

## Results

### Active Turnover of Heme via *de novo* Synthesis and Degradation During Pre-hibernation and Starvation to Protect Cells From Toxic Free Heme

**FIGURE 1:**
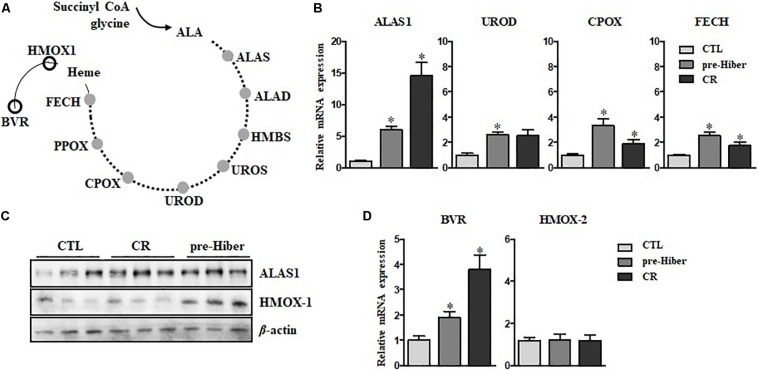
Active turnover of heme *via de novo* synthesis and degradation during pre-hibernation. **(A)** This scheme depicts the eight enzymes involved in *de novo* heme synthesis. **(B)** Quantitative RT-PCR was performed to estimate the relative levels of ALAS1, UROD, CPOX, and FECH mRNA in pre-hibernation as well as starvation. **(C)** The expression of ALAS1 and HMOX-1 was measured by western blotting. **(D)** The mRNA levels of BVR and HMOX-2 in the liver tissue during pre-hibernation and starvation were measured using RT-PCR and compared to those in the control. β-actin served as the loading control. Data are representative of 3 independent experiments and are expressed as mean ± SD, ^∗^*p* < 0.05 compared the group.

The heme biosynthetic pathway consists of eight enzymes (Lee et al., 2014). The first rate-limiting step occurs in the mitochondria where succinyl-CoA and glycine are converted into δ-aminolevulinic acid (ALA) by aminolevulinic acid synthase (ALAS). The remaining seven enzymes work sequentially to catalyze a series of chemical reactions that ultimately lead to the formation of heme (Figure 1A). These enzymes are ALA dehydratase (ALAD), hydroxymethylbilane synthase (HMBS), uroporphyrinogen III synthase (UROS), uroporphyrinogen decarboxylase (UROD), coproporphyrinogen oxidase (CPOX), protoporphyrinogen oxidase (PPOX), and ferrochelatase (FECH). Among these, upregulation of the ALAS, UROD, CPOX, and FECH mRNAs was consistently observed during pre-hibernation (Figure 1B).

Once synthesized, heme is incorporated into heme binding proteins to form hemoproteins, which regulate a variety of biological processes (Ajioka et al., 2006). While heme is essential for the functions of hemoproteins, free heme is toxic. The level of intracellular heme is determined by HO-1, the rate-limiting enzyme for heme degradation, catalyzing the first reaction of heme turnover, and generating biliverdin, iron, and carbon monoxide (Figure 1A). Biliverdin reductase (BVR) then reduces the central methene bridge of biliverdin, producing bilirubin. Similar to the results obtained in previous studies (Ni and Storey, 2010), the expression of HO-1 was augmented in hibernating animals (Figure 1C). Also, upregulation of BVR mRNA was observed, while the levels of HO-2 mRNA levels did not get altered in pre-hibernation (Figure 1D). We assessed the levels of heme biosynthetic enzymes in animals under the conditions of starvation and pre-hibernation, as pre-hibernation routinely consists of both hypothermia and starvation. Interestingly, the mRNA levels of the enzymes, ALAS1, UROD, CPOX, and FECH were dramatically increased in the torpid period of pre-hibernation compared to that in the control (Figure 1B). The calorie-restricted animals also showed significant increases in mRNA expression of these enzymes. However, the increase in the expression of UROD, CPOX, and FECH, was more pronounced in pre-hibernation than during starvation. Although heme biosynthesis was high for both conditions, these results suggest that, during pre-hibernation, heme biosynthesis is more robust and that heme has a more rapid turnover.

### Upregulation of Heme, but Not Reducing Hemoglobin, in Liver

**FIGURE 2:**
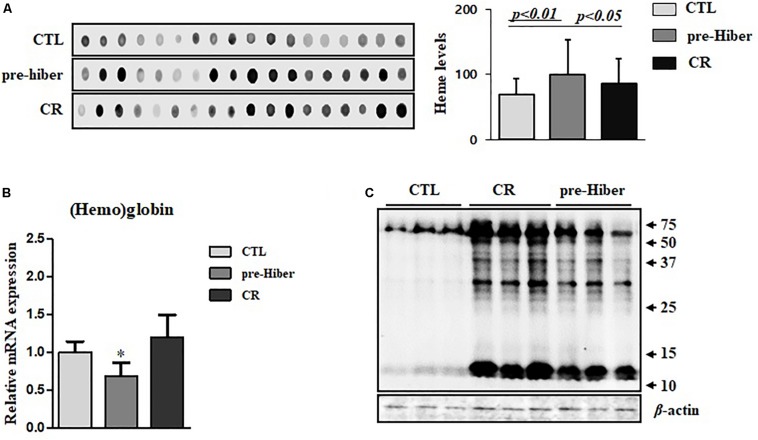
The cellular level of heme and hemoglobin during pre-hibernation. **(A)** Heme levels were measured using a dot blot assay. To measure heme level, the samples from different mice were extracted and a dot blot assay was performed. **(B)** Hemoglobin levels were detected by quantitative RT-PCR. Western blotting was performed for the measurement of hemoproteins. **(C)** Hemoproteins were classified based on biological function. β-actin served as the loading control. Data are representative of 3 independent experiments and are expressed as mean ± SD, ^∗^*p* < 0.05 compared the group.

To further investigate heme biosynthesis, we analyzed heme levels in liver tissues by dot blot analysis. This analysis showed a pronounced increase in heme levels in animals maintained in conditions of starvation as well as pre-hibernation, relative to that in controls (Figure 2A). However, we observed that the heme level in hemoglobin of these animals was significantly reduced, compared to that in the controls (Figure 2B), whereas the expression of heme protein, as measured by western blot, was still high (Figure 2C). Collectively, these results suggest that heme metabolism was more robust during the inactive state in these animals, compared to their normal, active state. By *in silico* analysis, we obtained an estimate of the possible biological processes of hemoproteins in the organisms, and about 6.4% of heme-containing proteins were noted to be involved in the metabolism (Supplementary Figure S1).

### Regulation of Heme-Iron Recycling During Acute Hibernation

**FIGURE 3:**
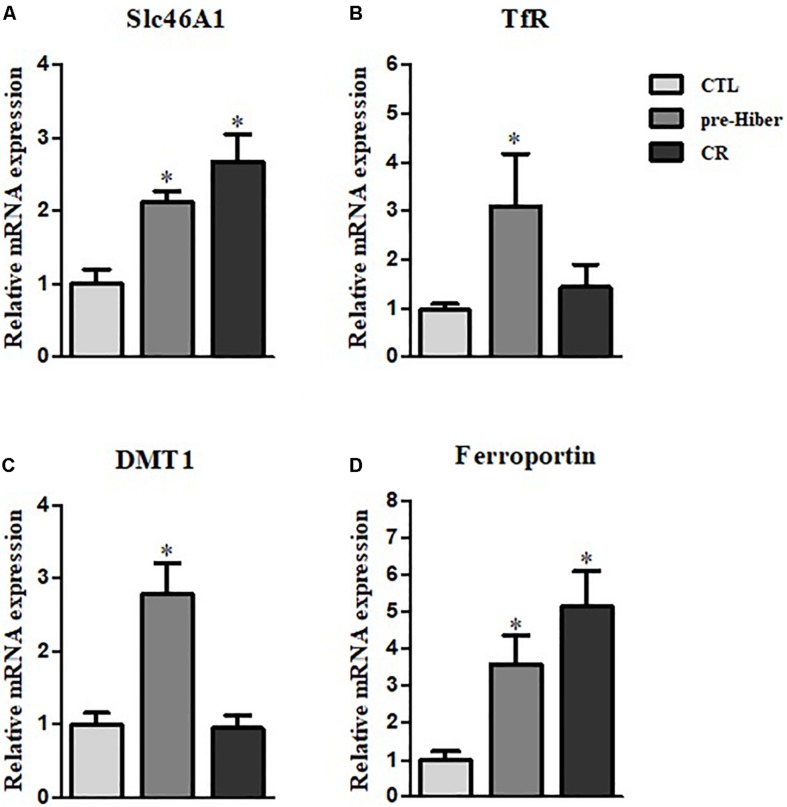
Regulation of heme-iron recycling during acute hibernation. **(A–D)** The mRNA levels of Slc46A1, TfR, DMT, and ferroportin were measured in the liver tissue of animals that had been maintained under conditions of pre-hibernation and starvation. β-actin served as the loading control. Data are representative of 3 independent experiments and are expressed as mean ± SD, ^∗^*p* < 0.05 compared the group.

To determine whether heme-iron recycling was increased in hibernating and starved Syrian hamsters, we evaluated the expression of heme-iron transporter and exporter mRNA levels under both conditions. As expected, the levels of SLC46A1, a transporter of heme, were increased in both of the above conditions, relative to that in the control (Figure 3A). As the cells ingest Tf-bound iron, via the TfR present on the cell surface, during pre-hibernation, the mRNA levels of TfR are increased (Figure 3B). The mRNA levels of DMT1 – which transports ferrous iron into cells and is necessary for intestinal uptake of inorganic sources of dietary iron – were also increased in pre-hibernation animals (Figure 3C). No increase was observed in the levels of TfR and DMT1 mRNA in the starvation group. The mRNA levels of the iron exporter, ferroportin, were higher in both pre-hibernation and starvation animals (Figure 3D). This indicates that these transporters are essential for heme-iron to reach its intracellular destinations, which enables its incorporation into various target proteins that carry out biological functions essential for survival during inactive periods. In summary, these results indicate that iron recycling was extensive during both pre-hibernation and starvation. The transport of iron is also a safety measure. High levels are toxic to cells, as an excess generates superoxide anions and hydroxyl radicals. For these reasons, iron recycling is tightly regulated among hibernators.

### Upregulation of Cytoprotective Proteins During Acute Hibernation

**FIGURE 4:**
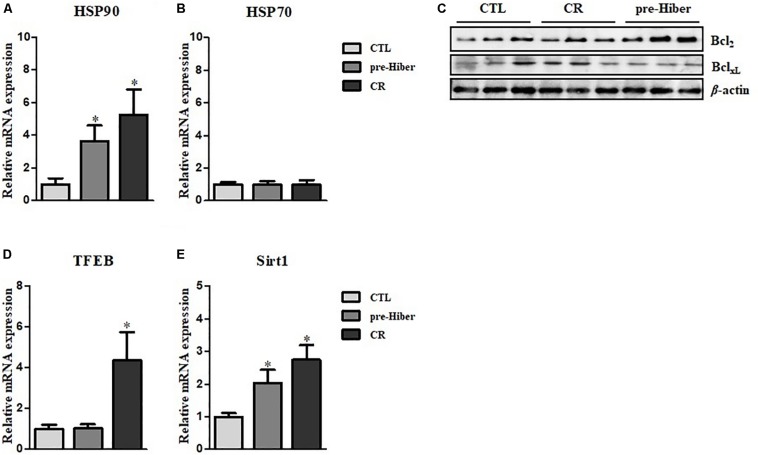
Measurement of HSP90, HSP70, TFEB, and Sirt1 level. **(A,B)** The mRNA levels of heat shock protein (HSP)90 and HSP70 in the tissue of pre-hibernation and calorie-restricted groups were evaluated by quantitative RT-PCR. **(C)** The expression of Bcl_2_ and Bcl_XL_ in liver was measured in hypothermic and calorie-restricted animals by western blotting. **(D)** The levels of TFEB mRNA were measured by quantitative RT-PCR. **(E)** The mRNA levels of Sirt-1 were analyzed by quantitative RT-PCR. Data are representative of 3 independent experiments and are expressed as mean ± SD, ^∗^*p* < 0.05 compared the group.

Hibernation seems to protect against cellular damage and promote wound healing, but the mechanisms underlying this cytoprotective effect are largely unknown (Otis et al., 2017). We observed increased expression of cytoprotective HO-1 in animals during pre-hibernation (Figure 1C). Additionally, we examined changes in the expression of other cytoprotective proteins, including heat shock proteins (HSP), HSP70 and HSP90, and the apoptosis pathway-related proteins, Bcl_2_ and Bcl_XL_. Messenger RNA and protein expression of HSP90 were increased both under pre-hibernation and starvation conditions in Syrian hamster liver (Figures 4A,B). Expression of the anti-apoptotic, Bcl_2_,was also increased in pre-hibernation, but not in starvation (Figure 4C). TFEB and Sirt-1 are known to act as cellular metabolic sensors. Upregulation of these proteins activates the cytoprotective mechanisms against starvation (Qin et al., 2006; Escobar et al., 2019). Thus, we assumed that the levels of these proteins would be increased in pre-hibernation, as this condition is accompanied by calorie restriction (CR). However, Sirt-1 mRNA, but not TFEB mRNA, was upregulated in pre-hibernation, whereas both Sirt-1 and TFEB mRNA were upregulated upon CR (Figures 4D,E).

## Discussion

Pre-hibernation imposes a stress similar to caloric restriction. In this study, pre-hibernation was compared to caloric restriction. Differential responses were observed in animals exposed to these conditions, suggesting that the physiological mechanisms in mammals exposed to pre-hibernation are different from those exposed to other stress inducers. In particular, HO was found to be upregulated in pre-hibernation animals. This enzyme catalyzes the first step of heme degradation in ground squirrels, thereby producing biliverdin/bilirubin, ferrous iron, and carbon monoxide (Ni and Storey, 2010). Of these products, carbon monoxide is able to activate protective mechanisms against stresses. In this context, it was important to analyze the metabolic processes of heme. However, to date, no study has explored.

Our study first determined that heme homeostasis was regulated in the pre-hibernation period in Syrian hamsters. We found that the heme-degradative enzyme, HO-1, was upregulated in hamsters during acute hibernation. HO-1 protects cells from harm by catabolically degrading excessive levels of heme. We also observed increased heme-iron recycling during starvation and pre-hibernation, and identified an increase in mRNA levels of Sirt-1. The induction of ALAS1 activity and heme levels were observed in the livers of fasting rats and mice. The homeostasis of heme is tightly regulated via controlled expression of ALAS1 and that of the heme-catabolizing enzyme heme, HO-1 (Unno et al., 2007; Wu et al., 2009). The induction of heme-anabolizing enzymes, such as ALAS-1, UROD, CPOX, and FECH, was observed in this study. Consequently, high heme levels suppressed ALAS1, and upregulated HO-1, *via* heme-sensitive regulatory proteins. Our finding of higher levels of ALAS-1 and HO-1 proteins in liver tissues confirmed the regulation of the heme levels by these enzymes. Remarkably, increased expression of HO-1 occurs in response to a wide variety of stimuli, such as heat shock, proinflammatory cytokines, and altered redox homeostasis. This provides increased cytoprotection under various cellular stress conditions (Pae et al., 2008). In our analysis, no change was observed in the expression of the constitutive form of this enzyme, HO-2.

Additionally, catabolism of heme releases bioactive byproducts, including iron which is readily bound by ferritin; CO which has anti-proliferative and anti-inflammatory properties; biliverdin which is reduced to anti-oxidative and potentially toxic and converted to bilirubin by BVR (Baranano et al., 2002; Ollinger et al., 2007). The increased expression of BVR further supports the increase in heme degradation. Increased levels of HO-1 may degrade the heme in the hemoglobin and result in its reduction during metabolic slowdown in hibernation. Moreover, we also detected high levels of heme transporters and ferroportin, suggesting that heme-iron allocation is likely critical to maintaining homeostasis in hibernating hamsters. Furthermore, these data suggest that heme metabolism and heme-iron recycling are essential for protecting cells under conditions of pre-hibernation and starvation.

Sirt-1 has been shown to promote adaptation to caloric restriction by regulating gluconeogenesis and glycolysis in the liver (Rodgers et al., 2005). In animals, starvation results in the binding of Sirt-1 with PPAR-γ DNA-binding sites and subsequent repression of target genes that drive the storage of fat (Tamori et al., 2002). Recently, it has been demonstrated that Sirt-1 regulates gene expression in white adipose tissue, similar to that in brown fat, modifying energy expenditure and adiposity in this tissue (Tamori et al., 2002). Hence, the direct link between Sirt-1 and metabolic modulation by HO-1 activity requires further investigation.

In summary, we have described heme catabolism during hibernation. Our study analyzed the regulation of molecules that are crucial for heme metabolism. The study also offers a potential new strategy for better regulation of intracellular heme concentrations during hypothermia and other stresses. Indeed, it is known that hypothermia can provide defense against accidental or surgically induced tissue damage. This study provides evidence for considering heme modulation as a means of better understanding the physiological processes of altered metabolic states, and also for designing more effective therapeutic strategies for various stress conditions by mimicking the hibernating state.

## Data Availability Statement

Sequencing data for mRNAs have been deposited to Sequence Syrian golden hamster (*Mesocricetus auratus*) at DNA Data Bank of Japan under accession number PRJDB9086.

## Ethics Statement

All experiments were performed in accordance with the ethical guidelines established by the Ethics Review Committee for animal experiments, and all animal work was approved by the Ethical Committee for Animal Research and all efforts were made to minimize the number of animals used in the course of this experiment.

## Author Contributions

HK designed the experiment and analyzed the data. PL performed all the experiments and wrote the manuscript. HK, NC, and HY analyzed the data and performed the data acquisition. NC and HY revised the manuscript.

## Conflict of Interest

The authors declare that the research was conducted in the absence of any commercial or financial relationships that could be construed as a potential conflict of interest.
