# Supplementary material for: Active Turnover of Heme in Hibernation Period in Mammals
Source: Front Physiol. 2020 Jan 15;10:1586. doi: 10.3389/fphys.2019.01586 (PMC6974447; doi:10.3389/fphys.2019.01586)
Supplement: Supplementary file 1 [file Data_Sheet_1.DOCX]

Supplementary Material

## Supplementary Figures

##
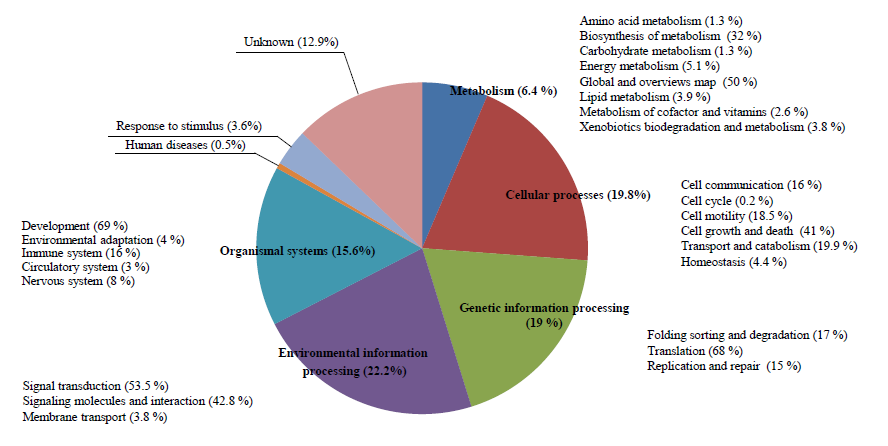


## Supplementary Figure 1. The possible biological processes of hemoproteins in the organisms was estimated using *in silico* model
